# Laparoscopic approach for intravesical surgery using pneumovesicum in the management of anterior colporrhaphy mesh erosion and stones around the bladder neck

**DOI:** 10.1590/S1677-5538.IBJU.2018.0046

**Published:** 2019-04-01

**Authors:** Young Dae Bae, Hoon Choi, Jae Hyun Bae, Bum Sik Tae

**Affiliations:** 1 Department of Urology, Korea University Ansan Hospital, Korea University College of Medicine, Korea

## Abstract

**Introduction and objective:**

Perforation of the bladder or urethra and erosion of the mesh after cystocele repair surgery are not uncommon and have potentially serious complications. Traditionally, surgical management of such complications has involved excision of the mesh using either a transurethral approach or open surgery. In this video, we present our experience of laparoscopic transvesical surgery for exposed mesh and stone.

**Materials and methods:**

Patient was placed in the lithotomy position under general anesthesia and a 30° operating cystoscope was inserted under direct vision. After filling the bladder with 300 mL normal saline, a 5 - mm VersaStep™ bladeless trocar was placed 2 cm above the pubic symphysis. Two more 5 mm trocars were placed bilaterally at 3 cm intervals from the initial trocar site. The pneumovesicum state was maintained at 8 - 12 mmHg and a 5 mm telescope was introduced. Using a curved dissector and curved Mayo scissors, the exposed mesh was mobilized and removed. Interrupted 4 - 0 Vicryl sutures were used to close the defect. To localize the ureteral orifice, intravenous Indigo Carmine was used. The bladder stones were removed through the urethra using a stone basket, guided using a ureteral stent pusher.

**Results:**

Total operation time was 55 min and the Foley catheter was removed at post - operative day 5 after postoperative cystography.

**Conclusions:**

Excellent visualization of mesh exposure and ureteral orifice was possible under laparoscopic transvesical surgery, and reconstruction including the mucosa and muscle layer was able to be achieved. This method is useful and feasible, with minimal invasiveness and an early post - operative recovery.

## ARTICLE INFO

**Available at: http://www.intbrazjurol.com.br/video-section/20180046_Bae_et_al**

**Int Braz J Urol. 2019; 45 (video #7): 410-410**

